# A Study on the Performance of Gel-Based Polyurethane Prepolymer/Ceramic Fiber Composite-Modified Asphalt

**DOI:** 10.3390/gels11070558

**Published:** 2025-07-20

**Authors:** Tengteng Guo, Xu Guo, Yuanzhao Chen, Chenze Fang, Jingyu Yang, Zhenxia Li, Jiajie Feng, Hao Huang, Zhi Li, Haijun Chen, Jiachen Wang

**Affiliations:** 1 School of Civil Engineering and Transportation, North China University of Water Resources and Electric Power, Zhengzhou 450045, China; guotth@ncwu.edu.cn (T.G.); 17538255803@163.com (X.G.); fangchenze@ncwu.edu.cn (C.F.); yangjingyu@ncwu.edu.cn (J.Y.); zhenxiali2009@ncwu.edu.cn (Z.L.); 18027459877@163.com (J.F.); 15951287427@163.com (H.H.); 15076861657@163.com (Z.L.); chenhaijun@ncwu.edu.cn (H.C.); 2 Technology Innovation Center of Henan Transport Industry of Utilization of Solid Waste Resources in Trafffc Engineering, North China University of Water Resources and Electric Power, Zhengzhou 450045, China; 3 Henan Province University-Enterprise Research and Development Center for Green, Low-Carbon and High-Performance Road Materials, Zhengzhou 450045, China; 4 Henan Province Engineering Technology Research Center of Environment Friendly and High-Performance Pavement Materials, Zhengzhou 450045, China; 5 Department of Electrical Engineering, College of Science and Technology, University of Nottingham Ningbo China, Ningbo 315100, China; ssyjw19@nottingham.edu.cn

**Keywords:** gel material, polyurethane prepolymer, ceramic fiber, rheological properties

## Abstract

In order to solve various problems in traditional roads and extend their service life, new road materials have become a research hotspot. Polyurethane prepolymers (PUPs) and ceramic fibers (CFs), as materials with unique properties, were chosen due to their synergistic effect: PUPs provide elasticity and gel-like behavior, while CFs contribute to structural stability and high-temperature resistance, making them ideal for enhancing asphalt performance. PUPs, a thermoplastic and elastic polyurethane gel material, not only enhance the flexibility and adhesion properties of asphalt but also significantly improve the structural stability of composite materials when synergistically combined with CF. Using response surface methodology, an optimized preparation scheme for PUP/CF composite-modified asphalt was investigated. Through aging tests, dynamic shear rate (DSR) testing, bending rate (BBR) testing, microstructure scanning (MSCR), scanning electron microscopy (SEM), atomic force microscopy (AFM), and infrared spectroscopy (IR), the aging performance, rheological properties, permanent deformation resistance, microstructure, and modification mechanism of PUP/CF composite-modified asphalt were investigated. The results indicate that the optimal preparation scheme is a PUP content of 7.4%, a CF content of 2.1%, and a shear time of 40 min. The addition of the PUP and CF significantly enhances the asphalt’s aging resistance, and compared with single-CF-modified asphalt and base asphalt, the PUP/CF composite-modified asphalt exhibits superior high- and low-temperature rheological properties, demonstrating stronger strain recovery capability. The PUP forms a gel network structure in the material, effectively filling the gaps between CF and asphalt, enhancing interfacial bonding strength, and making the overall performance more stable. AFM microscopic morphology shows that PUP/CF composite-modified asphalt has more “honeycomb structures” than matrix asphalt and CF-modified asphalt, forming more structural asphalt and enhancing overall structural stability. This study indicates that the synergistic effect of PUP gel and CF significantly improves the macro and micro properties of asphalt. The PUP forms a three-dimensional elastic gel network in asphalt, improving adhesion and deformation resistance. Using response surface methodology, the optimal formulation (7.4% PUP, 2.1% CF) improves penetration (↓41.5%), softening point (↑6.7 °C), and ductility (↑9%), demonstrating the relevance of gel-based composites for asphalt modification.

## 1. Introduction

Traditional asphalt pavement is prone to typical problems such as rutting, cracking, and potholes in high-temperature environments, especially due to heavy-duty vehicles and frequent traffic loads [1,2,3,4,5]. The accelerated degradation of pavement structural performance will not only lead to a substantial decrease in the service life of roadways but will also result in a significant rise in maintenance and repair costs. In order to address this issue, researchers have conducted experiments to assess the combinations and proportions of various modifiers, aiming to evaluate their effects on asphalt performance. This study seeks to develop tailored modified asphalt solutions that cater to specific engineering requirements. Some scholars have found that adding fiber to asphalt mixture can significantly enhance the mechanical properties of the mixture and effectively improve its performance under high- and low-temperature conditions. This method also enhances the mixture’s water stability and fatigue resistance, thereby extending the service life of asphalt pavement and reducing maintenance costs [6,7,8,9,10,11,12].

Polyurethane, an organic polymer composed of hard segments (isocyanates) and soft segments (polyols), where the former enhances rigidity and the latter imparts good toughness, and gel materials—colloidal or polymeric systems with solid-like behavior due to three-dimensional network structures whose ability to encapsulate particles and adjust mechanical behavior makes them critical in asphalt design, particularly for enhancing elasticity and durability—are both key components in this context. [13,14,15,16,17,18,19]. PUP contains reactive -NCO groups that, during mixing, react with hydroxyls in asphalt to form a urethane network. This crosslinked gel structure enhances elasticity, fills voids, and bridges weak interfacial regions between fibers and asphalt, improving deformation resistance [20,21,22]. As a crucial intermediate in the synthesis of polyurethane materials, polyurethane prepolymer (PUP) is synthesized through the reaction of R-N=C=O groups with active hydrogen-containing compounds. During the reaction, the N=C double bond in the isocyanate group is broken, and active hydrogen is transferred to the N atom, ultimately forming a urethane structure (R-NHCOO) [23,24,25,26,27,28,29]. Akimov, Liu et al. [30,31] added polyurethane prepolymer into matrix asphalt at 140 °C through high-speed shearing to prepare polyurethane-modified asphalt. Experimental analyses showed that the anti-aging performance of the modified asphalt was improved, and its stability was good. Infrared spectroscopy confirmed that the isocyanate groups in polyurethane react with the hydroxyl groups in asphalt to form new chemical bonds through group bonding. Fang Ying et al. [32] determined the optimal parameters for preparing polyurethane prepolymer-modified asphalt, finding that its performance is best at high temperature but its temperature sensitivity is strongest at 52–82 °C. Cuadri et al. [33] prepared polyurethane-modified asphalt by using castor oil instead of polyols to react with diisocyanates, and the results showed that the rheological properties and rutting resistance of castor oil-based polyurethane-modified asphalt were both improved. Vural Kök et al. [34] synthesized polyurethane derivatives by polymerizing 4,4′-diphenylmethane diisocyanate with novel modified palm oil-based polyols to modify asphalt. The study showed that when the curing condition was 24 h at 85 °C (compared with 1 h), the rutting factor of the asphalt increased by 256%, and the optimal modification process parameters were determined by optimizing the curing time, temperature, NCO/OH molar ratio, and PUP content. Rabindra K Padhan et al. [35] found that in situ polymerization enables better dispersion of polymers in asphalt, and a new type of polyurethane-modified asphalt synthesized in situ with terephthalamide and MDI in asphalt improved the low-temperature, high-temperature, and fatigue properties of the asphalt.

Ceramic fiber, a fibrous material made of ceramic materials, is characterized by its light weight, high strength, high temperature resistance, low thermal conductivity, good chemical stability, and excellent thermal insulation performance [36,37]. It has gradually attracted attention in the asphalt field: its low thermal conductivity can reduce heat accumulation in asphalt pavements, delay asphalt softening, and reduce the risk of high-temperature rutting; its chemical stability can slow down asphalt aging and extend pavement service life. WAN Jiuming et al. [38] used DSR to confirm that ceramic fibers can significantly enhance the high-temperature performance of asphalt, and scanning electron microscopy (SEM) and atomic force microscopy (AFM) observations showed that ceramic fibers have good dispersibility in asphalt. Arabani M, Shabani A et al. [39,40] found that adding ceramic fibers to asphalt can improve its high-temperature performance, but its low-temperature performance decreases slightly; additionally, ceramic fibers can enhance the permanent deformation resistance and fatigue life of asphalt mixtures, with a recommended dosage of 3%. Zhou Haicheng et al. [41,42] found that the addition of ceramic fibers significantly enhances the high-temperature performance of asphalt mixtures but also causes a slight reduction in low-temperature performance. Xiao Ziwei [43] found that nano-AAT/ceramic fiber composite-modified asphalt, although with slightly weakened low-temperature performance, has excellent high-temperature deformation resistance and shows significant advantages in pavement performance, including high-temperature stability, low-temperature crack resistance, and water stability.

In summary, it is evident that different fibers can significantly enhance specific properties of asphalt and asphalt mixtures. Ceramic fibers, known for their exceptional high-temperature and corrosion resistance, can markedly improve the high-temperature stability, mechanical strength, and fatigue resistance of asphalt, thereby effectively extending the service life of pavements. However, their improvement effect on asphalt’s low-temperature performance is limited, and when the fiber dosage exceeds a certain amount, it may instead reduce the low-temperature crack resistance of asphalt. A common problem with fiber-modified asphalt is the poor dispersibility of fibers in asphalt, which makes it prone to agglomeration during mixing and affects the modification effect. Ceramic fibers have highly inert surfaces and weak compatibility with asphalt, making them prone to segregation during storage and possibly causing shedding during long-term use. Surface modification methods, such as NaOH alkali treatment and silane coupling agents (KH-550), have been proposed and used in this study to improve fiber dispersion and chemical compatibility with asphalt.

## 2. Results and Discussion

### 2.1. The Best Preparation Scheme of PUP/CF Composite-Modified Asphalt

#### 2.1.1. Analysis of Response Surface Method Test Results

According to the level of influencing factors, 17 sets of test schemes corresponding to the response indicators were designed and generated. As shown in Table 1, in order to ensure the reliability and stability of the model and reduce the model error, five repeated tests were carried out at the center point of the parameter design.

**Figure 1:**
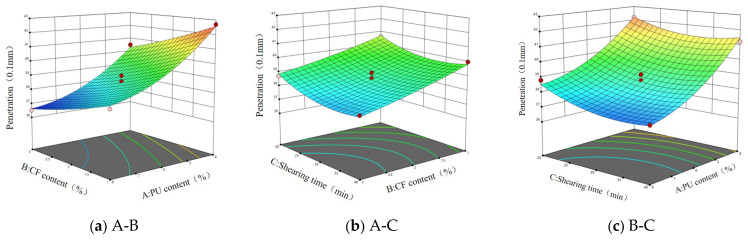
Response surface of three factors to penetration.

**Figure 2:**
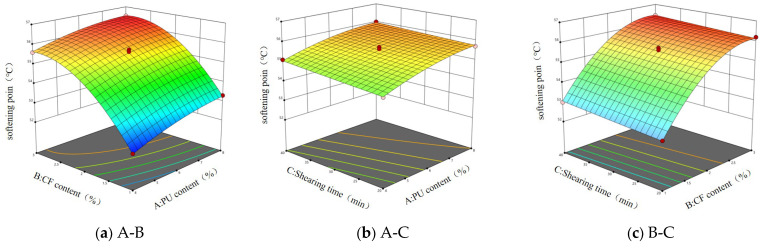
Response surface of three factors to softening point.

**Figure 3:**
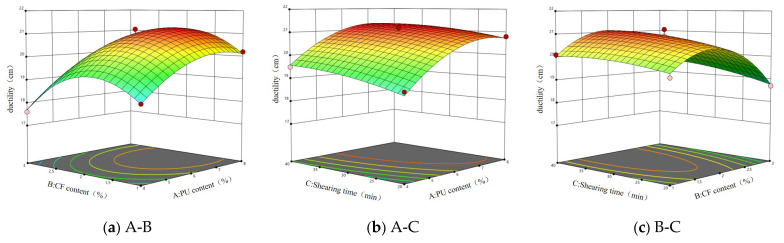
Response surface of three factors to ductility.

**Figure 4:**
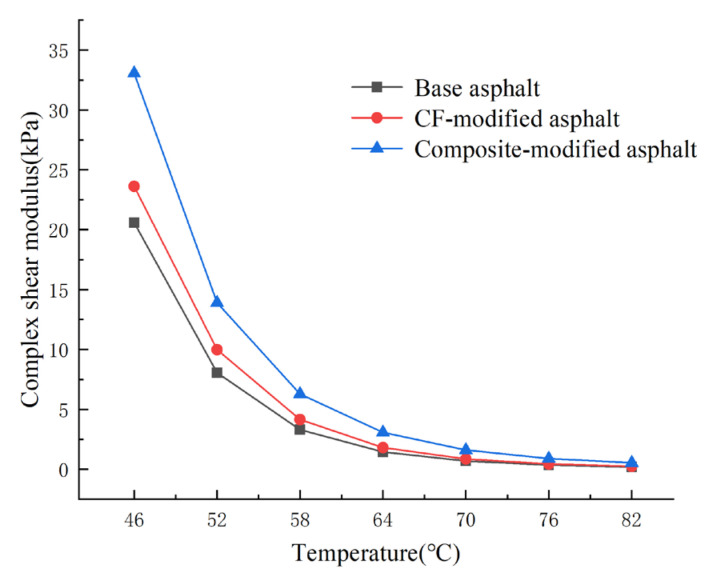
Complex modulus test results.

According to the data in Table 1, multiple regression analysis was carried out, and A (polyurethane prepolymer content), B (ceramic fiber), and C (shear time) were used as response surface variables. The response surface models of penetration, softening point, and ductility were established by using the software Design-Expert 13 and were expressed as (1)~(3), respectively. The response surface diagrams of the influencing factors affecting the response value are shown in Figure 1, Figure 2, Figure 3 and Figure 4.

(1) $$\begin{array}{l} X=38.6-0.9875A-1.99B-0.5C+0.15AB-0.075AC-0.125BC+0.125A^{2}+0.725B^{2}\\ +0.55C^{2} \end{array}$$

(2) $$\begin{array}{l} X=55.54+0.5125A+1.7B+0.1625C-0.075AB-0.05AC-0.025BC-0.0195A^{2}-0.97B^{2}\\ +0.005C^{2} \end{array}$$

(3) $$\begin{array}{l} X=20.94+0.8875A-0.325B+0.3125C+0.075AB-0.1AC+0.125BC-0.72A^{2}-1.25B^{2}\\ -0.17C^{2} \end{array}$$

It can be seen from Figure 1 that when the shear time is constant, an increase in polyurethane prepolymer content and ceramic fiber content will make the penetration decrease, but an increase of just the polyurethane prepolymer content will make the slope of the response surface steeper, which indicates that the increase in the polyurethane prepolymer has a significant effect on the penetration of asphalt. When the content of ceramic fiber is constant, the increase in polyurethane prepolymer content and shear time will make the penetration decrease. However, when A is 4%, with the increase in shear time, the penetration shows a trend of decreasing first and then increasing. The response surface in Figure 1c is approximately flat, which indicates that the comprehensive influence of ceramic fiber content and shear time on asphalt penetration is relatively stable.

It can be seen from the response surface in Figure 2a that with the increase in A, the softening point gradually increases, but the increase is small. When B is 1%, with the increase in A, the softening point increases from 52 °C to 53.2 °C. This shows that the addition of the polyurethane prepolymer can improve the softening point of asphalt, but the effect is relatively mild. It can be seen from Figure 2b that the response surface is approximately flat, which indicates that the combined effect of the polyurethane prepolymer content and shear time on the softening point of asphalt is relatively stable, and there may be no strong interaction between the two. From Figure 2c, it can be seen that with the increase in B, the response curve is steeper, and when the shear time changes, the softening point changes less, indicating that the influence of B on the softening point is more significant.

Based on the steepness of the surface in Figure 3a, the influence of factor B on the change in asphalt ductility is more significant than that of factor A, which indicates that the change in ceramic fiber content is more sensitive to the change in ductility. As can been seen by the similar trend of the two factors in relation to ductility change, it is speculated that there is a certain interaction between factor A and factor B. It can be seen from Figure 3b that the change in factor A has a more significant effect on the steepness of the surface, indicating that the polyurethane prepolymer content has a greater influence on the ductility of the asphalt. With the increase in the PUP content, the ductility of the asphalt increases first and then decreases. It can be seen from Figure 3c that there is an interaction effect of ceramic fiber content and shear time on the ductility of the response value. With the change in B, the surface is more steep, indicating that the influence of ceramic fiber content on asphalt ductility is more significant than that of shear time. When the shear time is constant, with the increase in ceramic fiber content, the ductility increases first and then decreases.

#### 2.1.2. Response Surface Optimization

According to the requirements of the relevant specifications of road asphalt, combined with the test purpose, a response surface optimization analysis was carried out. The lower the penetration of asphalt, the better the high-temperature stability, so the minimum penetration was used; the higher the softening point of asphalt, the stronger its high-temperature stability, so the maximum softening point was used. The greater the ductility of the asphalt, the stronger its low-temperature cracking resistance, so the maximum value of ductility was used. According to the performance requirements, the response surface method was used to optimize the performance, and the optimization results of the response value preparation parameters were obtained, as shown in Table 2.

The performance of asphalt preparation was optimized by the comprehensive response surface results, and the optimum preparation conditions of modified asphalt were determined as follows: the content of polyurethane prepolymer was 7.4%, the content of ceramic fiber was 2.1%, and the shear time was 40 min. These values were determined via RSM optimization and validated through experimental tests. The error between the predicted and actual values was less than 3%, confirming the statistical reliability of the model.

#### 2.1.3. Validation of the Best Dosage of Composite Modification

According to the optimized preparation parameters, the preparation of PUP/CF composite-modified asphalt was carried out, and the three indicators were tested. The test results were compared with the predicted values, as shown in Table 3.

It can be seen that the error between the measured value and the predicted value is small, not exceeding 3%. This shows that the model can well describe the relationship between variables and also reflects that the selection of factors and the setting of levels are more reasonable.

### 2.2. Aging Performance Analysis

After testing the matrix asphalt, 2% CF-modified asphalt and PUP/CF composite-modified asphalt were subjected to 85 min RTFOT; the changes in the three indexes of penetration, softening point, and ductility before and after aging were measured as shown in Table 4.

It can be seen from Table 4 that after aging, the penetration of the three kinds of asphalt showed a significant downward trend. Compared with the matrix asphalt, the residual penetration ratio of CF-modified asphalt increased by 2.9%, indicating that CF improved the anti-aging performance of asphalt. The residual penetration of PUP/CF composite-modified asphalt was 12.9% higher than that of CF-modified asphalt, indicating that the addition of PUP further improved the anti-aging performance. The softening point of the three kinds of asphalt increased after aging. The minimum increase in PUP/CF composite-modified asphalt was 1.7 °C, and the maximum increase in softening point of matrix asphalt was 2.9 °C. The addition of modifiers made the softening point increment decrease gradually, indicating that CF and PUP composite modification can improve the anti-aging performance of asphalt. The mass loss of matrix asphalt, CF-modified asphalt, and PUP/CF composite-modified asphalt was 0.348%, 0.152%, and 0.086%, respectively. The mass loss of PUP/CF composite-modified asphalt and CF-modified asphalt was reduced by 0.196% and 0.262%, respectively, compared with that of matrix asphalt, indicating that CF can effectively improve the anti-aging ability of asphalt and reduce the mass loss. The addition of PUP can effectively inhibit mass loss of asphalt and improve the anti-aging performance of asphalt.

### 2.3. Analysis of Dynamic Shear Rheological Test

#### 2.3.1. Temperature Scanning Analysis

**Figure 5:**
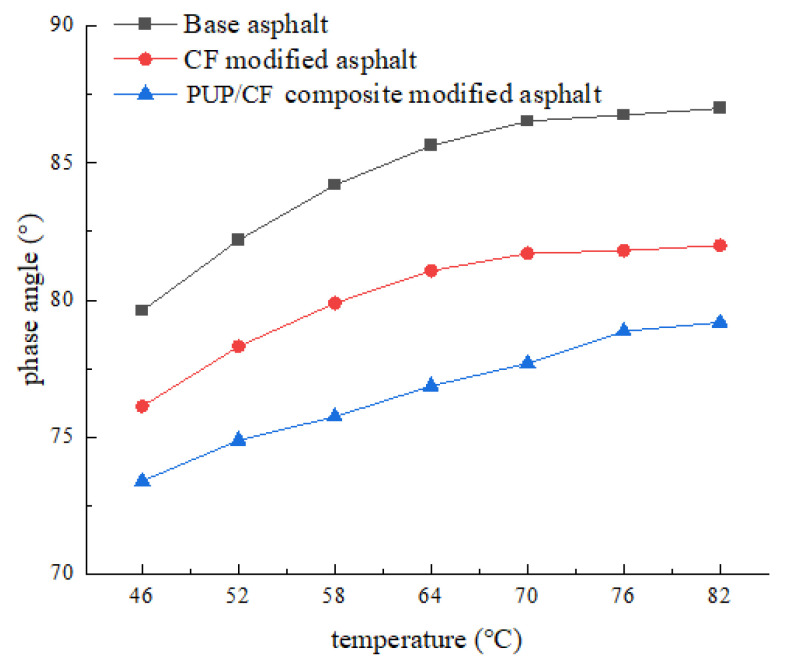
Phase angle test results.

The temperature scanning test results of PUP/CF composite-modified asphalt are shown in Figure 4 and Figure 5.

It can be seen from Figure 4 that the complex shear modulus of the three kinds of asphalt decreases with the increase in temperature. Under different temperature conditions, the complex shear modulus of PUP/CF composite-modified asphalt is the largest. On the whole, the complex shear modulus of the PUP/CF composite-modified asphalt is significantly higher than that of both the matrix asphalt and the CF-modified asphalt at low temperatures. However, as the temperature increases, the complex shear modulus gradually decreases and approaches the level of the matrix asphalt, although it remains consistently higher than that of the matrix asphalt. It can be seen from Figure 5 that the phase angle of the three kinds of asphalt increases with the increase in temperature. The δ of matrix asphalt increased from 79.62° to 86.99°, that of CF-modified asphalt increased from 76.13° to 82.0°, and that of PUP/CF-modified asphalt increased from 73.40° to 79.19°. The higher the temperature, the greater the viscosity of the asphalt, resulting in an increase in δ. The phase angle of PUP/CF-modified asphalt is the smallest at the same temperature.

**Figure 6:**
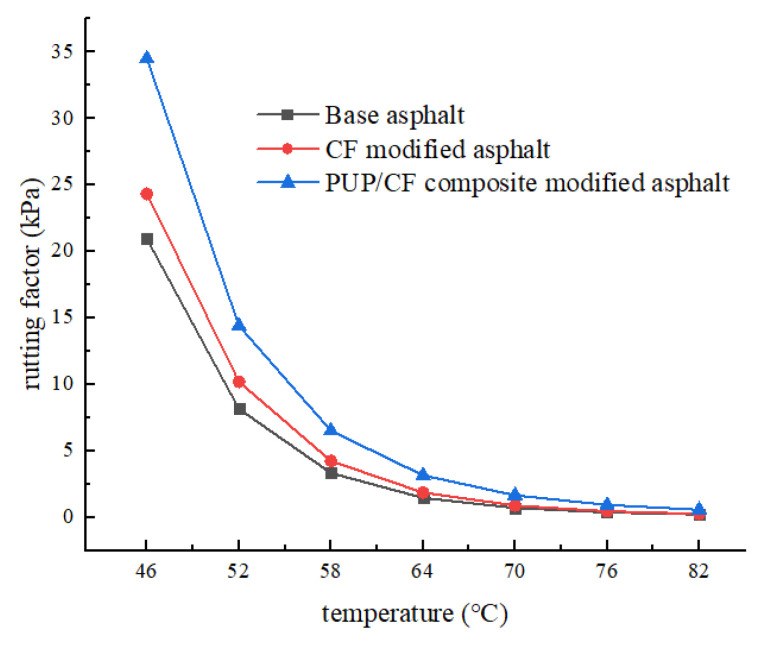
Rutting factor–temperature curve.

It can be seen from Figure 6 that the rutting factor decreases significantly with the increase of temperature, and the rutting factor of PUP/CF composite modified asphalt is the largest, followed by CF modified asphalt, and the matrix asphalt is the smallest. However, as the temperature rises to a certain extent, the gap between the three rutting factors gradually narrows. This is due to the transition of asphalt from an elastic state to a viscous state at high temperature. The viscous component increases, the elastic component decreases, and the asphalt is more likely to deform, so the rutting factor will decrease. At 46 °C, the maximum rutting factor of PUP/CF composite-modified asphalt is 34.50 kPa, which is 10.17 kPa larger than that of CF-modified asphalt and 13.57 kPa larger than that of matrix asphalt, indicating that CF plays a role of filling and supporting asphalt to prevent excessive deformation at high temperature. PUP can fill the gap between fibers, and its internal structure is more stable; so, the resistance to deformation of asphalt is improved, and the rutting factor increases.

#### 2.3.2. Frequency Scanning Analysis

(1) G* analysis of Different Asphalt Complex Moduli

**Figure 7:**
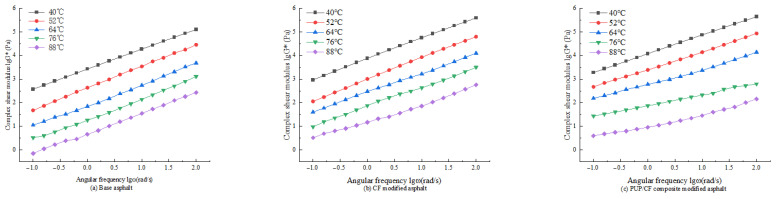
Asphalt lgG*-lg*ω* relationship diagram.

The variation in the complex modulus of asphalt with loading frequency at different temperatures was explored in detail. According to the test results, the logarithm was taken and the complex modulus (lgG*)–angular frequency (lgω) curve was drawn, as shown in Figure 7.

It can be seen from Figure 7 that the complex shear modulus of different asphalts increases with the increase in angular frequency at the same temperature, and the two show an approximate linear correlation. This is because when the angular frequency increases, the load action time is shortened, the elastic characteristics of the asphalt are highlighted, and the elastic deformation increases, making the complex shear modulus increase. The higher angular frequency corresponds to the high speed of the vehicle, and the complex shear modulus is the ability of the asphalt to resist deformation. Under the same temperature and the same frequency, the complex shear modulus of PUP/CF composite-modified asphalt is the largest, and the matrix asphalt is the smallest. This shows that the addition of PUP increases the elastic properties of asphalt, and CF increases the stiffness of asphalt. The combination of the two makes the asphalt show better anti-deformation ability.

(2) Time–temperature equivalent master curve construction and analysis

**Figure 8:**
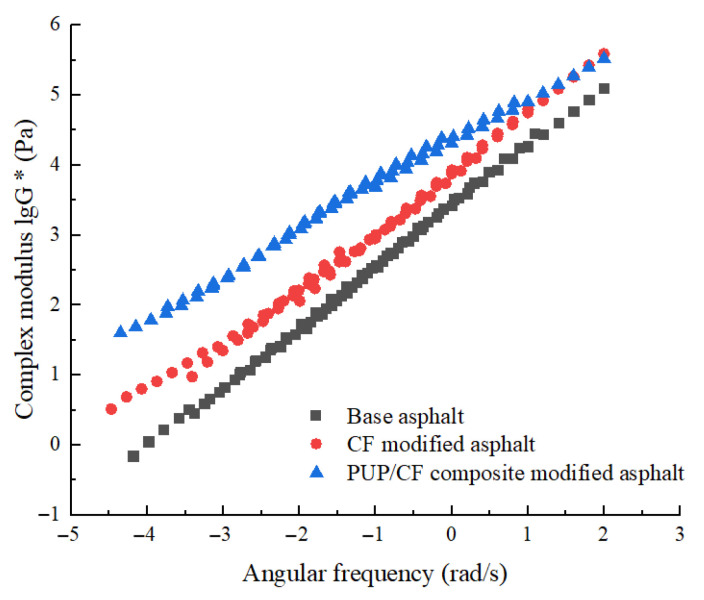
The main curves of lgG*-lg*ω* of asphalt.

By comparing the mechanical properties at different temperatures with those at the reference temperature, the shift factor of each temperature relative to the reference temperature was determined. According to the displacement factor, displacement was carried out, and the main curve of lgG*-lgω was obtained. Based on the displacement factor at the test temperature of 40 °C, the master curve of the composite modulus at the reference temperature of 40 °C was drawn, as shown in Figure 8.

According to the analysis of Figure 8, it can be concluded that under the condition of high temperature and low frequency, the complex shear modulus of matrix asphalt is the lowest, indicating that its high-temperature deformation resistance is weak; the complex shear modulus of CF-modified asphalt is slightly higher than that of matrix asphalt, and CF has good thermal stability. The complex modulus of PUP/CF composite-modified asphalt is significantly higher than that of CF-modified asphalt and matrix asphalt, indicating that the synergistic effect of PUP and CF can effectively enhance the high-temperature performance of asphalt. At low temperature and high frequency, as the angular frequency increases, the complex shear modulus of the three asphalts gradually increases. Among them, CF-modified asphalt gradually tends to PUP/CF composite-modified asphalt. PUP/CF composite-modified asphalt is the largest, because PUP has excellent flexibility and low-temperature performance and can improve the flexibility of asphalt in low-temperature environments. CF plays a role in enhancing the skeleton in asphalt, which greatly improves the overall strength and stiffness of asphalt. When PUP and CF are used to modify asphalt, PUP can fill the tiny voids that may exist between the CF and asphalt, enhance the bonding force between the fiber and asphalt, and make the synergy between them closer. They complement each other’s advantages, significantly improving the elastic properties of asphalt and enhancing its ability to resist deformation. Therefore, the addition of PUP and CF significantly improves the deformation resistance of asphalt.

### 2.4. Multi-Stress Creep Recovery Test

**Figure 9:**
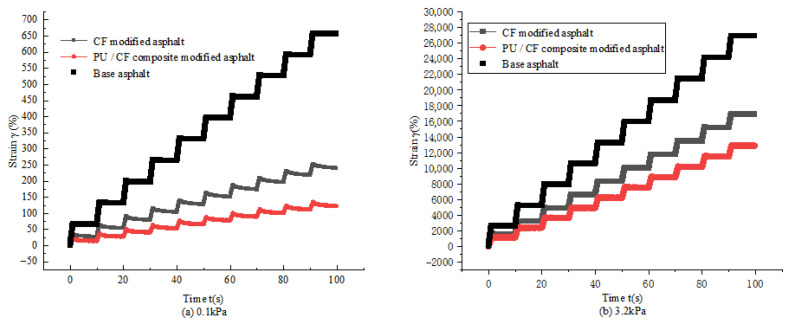
Strain variation with time under different stress levels.

The test results are shown in Figure 9. Under a creep cycle of 0.1 and 3.2 kPa stress levels, the strain is gradually increasing. It can be seen from Figure 9a,b that the strain level of the matrix asphalt in a cycle period shows almost no recovery ability, indicating that the matrix asphalt has relatively weak resistance to deformation when subjected to stress. It can be seen from Figure 9a,b that under the same stress level, the cumulative strain of PUP/CF composite-modified asphalt is lower than that of CF-modified asphalt. At 0.1 kPa, both asphalts have good recovery ability, but at 3.2 kPa, the recovery strain ability of the two modified asphalts becomes weaker; compared with the matrix asphalt, the cumulative strain is greatly reduced, indicating that PUP and CF can give asphalt better resistance to deformation and effectively inhibit strain accumulation under stress. All tests were performed in triplicate, and the results are reported as the mean ± standard deviation. One-way ANOVA (α = 0.05) was used to assess the significance of differences between modified and unmodified asphalt.

**Figure 10:**
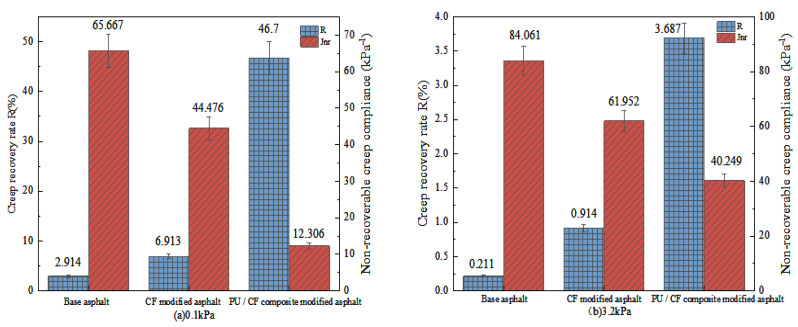
R and Jnr of three kinds of asphalt under different stress.

The data obtained from the test were used to calculate R and Jnr under different stresses, and the results are shown in Figure 10.

It can be seen from Figure 10 that under the stress of 0.1 kPa, the R value of matrix asphalt is the smallest, and the R value of CF-modified asphalt is not significantly higher than that of matrix asphalt, which is only increased by 3.999%. The R value of PUP/CF composite-modified asphalt is the largest, which is 43.786% higher than that of matrix asphalt. It shows that the addition of CF has no obvious effect on the strain recovery of asphalt. The chemical structure and physical properties of PUP enhance the elastic composition of CF-modified asphalt, improve the flexibility and elasticity of composite modified asphalt, and enhance the resistance to deformation of asphalt. It can be seen from (b) that compared with the stress of 0.1 kPa, the R of the three asphalts at the stress level of 3.2 kPa is greatly reduced. Under the action of large stress, their recovery deformation ability is weakened, but PUP/CF composite-modified asphalt have a relatively higher performance than CF-modified asphalt and matrix asphalt. The performance is the best, indicating that the addition of PUP and CF improves the creep recovery ability of asphalt, but under large stress, the improvement effect is weakened.

### 2.5. Bending Creep Stiffness Test Analysis

**Figure 11:**
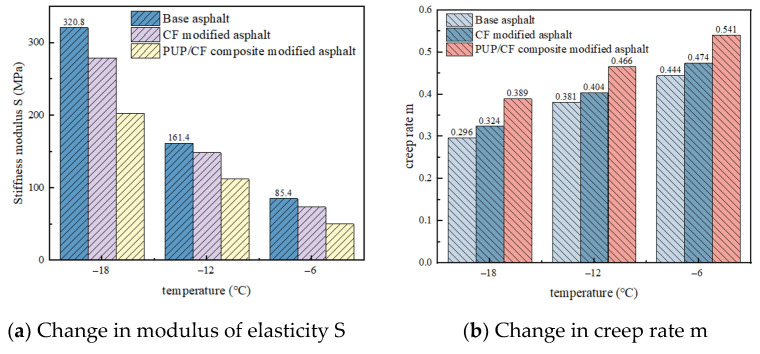
The variation in stiffness modulus and creep rate with temperature.

The results of the bending creep stiffness test are shown in Figure 11a. It can be seen that the stiffness modulus of matrix asphalt, CF-modified asphalt, and PUP/CF composite-modified asphalt increases with the decrease in temperature. At −6 °C, −12 °C, and −18 °C, CF-modified asphalt has a bending creep stiffness 11.8%, 13%, and 41.7% lower than that of matrix asphalt, and PUP/CF composite-modified asphalt has a bending creep stiffness 35.1%, 49.1%, and 118.4% lower than that of matrix asphalt. This shows that in a low-temperature environment, the effect of CF on the anti-deformation ability of asphalt is relatively limited. At low temperature, the brittleness of asphalt itself increases, and the reinforcement effect of CF struggles to offset the adverse effect of low temperature on asphalt performance, while the addition of PUP enhances the low-temperature performance of CF-modified asphalt. It can be seen from Figure 11b that the creep rate decreases with the decrease in temperature. At the three temperatures of −6 °C, −12 °C, and −18 °C, the creep rate of PUP/CF composite-modified asphalt is the largest, and that of matrix asphalt is the smallest. The creep rate of CF-modified asphalt increases by 0.03, 0.023, and 0.028, respectively, at the three temperatures, and the creep rate of PUP/CF-modified asphalt increases by 0.097, 0.085, and 0.093, respectively, compared with matrix asphalt. CF mainly relies on physical hindrance to reduce the creep rate, and the improvement in low-temperature creep performance of asphalt is limited. For PUP in PUP/CF composite-modified asphalt, its activity at low temperature is relatively strong, and the elastic effect of PUP still makes the creep rate of asphalt relatively high, so the low-temperature rheological properties of PUP/CF composite-modified asphalt are the best.

Compared with base asphalt, the PUP/CF composite modification significantly improves tensile strength (by ~25%), fatigue life (by ~30%), and high-temperature rutting resistance (by ~20%), while also enhancing interfacial bonding and microstructural uniformity.

### 2.6. Scanning Electron Microscope Test Analysis

**Figure 12:**
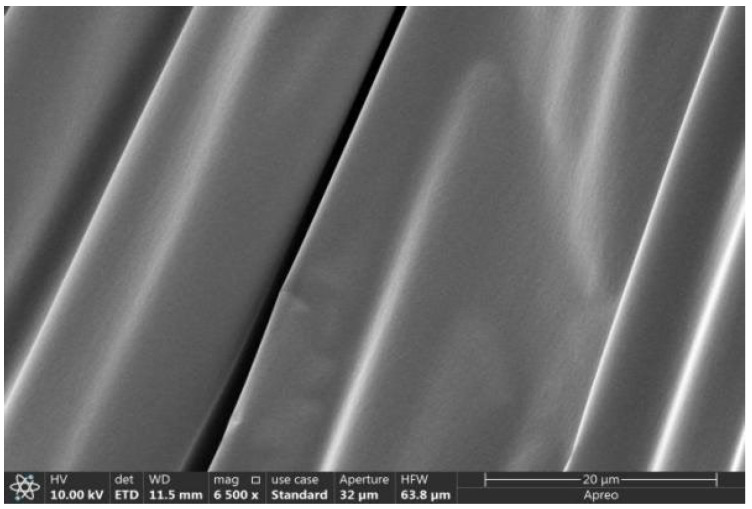
Microstructure of matrix asphalt.

**Figure 13:**
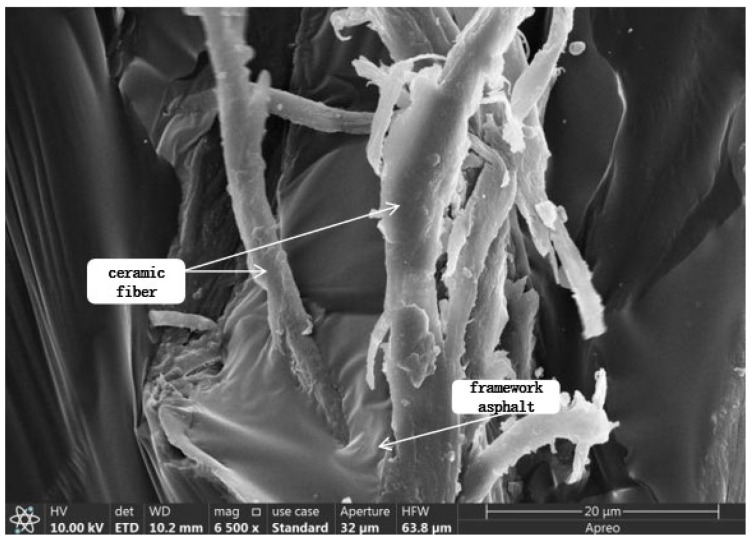
Microstructure of CF-modified asphalt.

**Figure 14:**
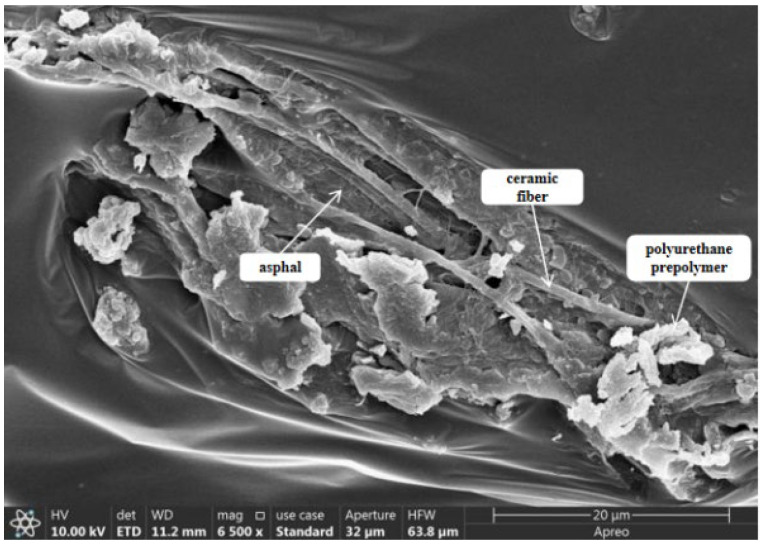
Microstructure of PUP/CF composite-modified asphalt.

The scanning electron microscope morphology of matrix asphalt, CF-modified asphalt, and PUP/CF composite-modified asphalt was studied, as shown in Figure 12, Figure 13 and Figure 14.

The analysis of Figure 12, Figure 13 and Figure 14 shows that the surface of the matrix asphalt is relatively smooth, the micro-morphology is evenly distributed, the surface is flat, and there is no obvious fluctuation and fracture. There are no impurities, holes, or cracks on the surface, indicating that the internal structure is dense and evenly distributed. In CF-modified asphalt, a large amount of asphalt is attached around CF, and an asphalt film is formed on the surface of the fiber, which can transform free asphalt into structural asphalt. When stressed, the force can be transferred from asphalt to CF, which enhances the mechanical properties of the whole material and makes asphalt more stable. CF shows a certain distribution state in asphalt, and the fibers are intertwined to form a three-dimensional network structure. Under the action of PUP, CF is closely combined with asphalt, and there is no obvious agglomeration phenomenon, indicating that CF and PUP have good dispersion in asphalt. The surface of CF is wrapped by PUP and asphalt, which enhances the interfacial bonding between CF and asphalt.

### 2.7. Atomic Force Microscope Test Analysis

**Figure 15:**
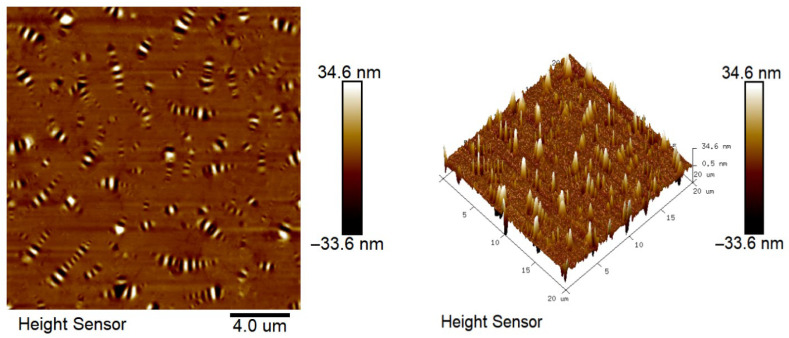
Microscopic morphology of matrix asphalt.

**Figure 16:**
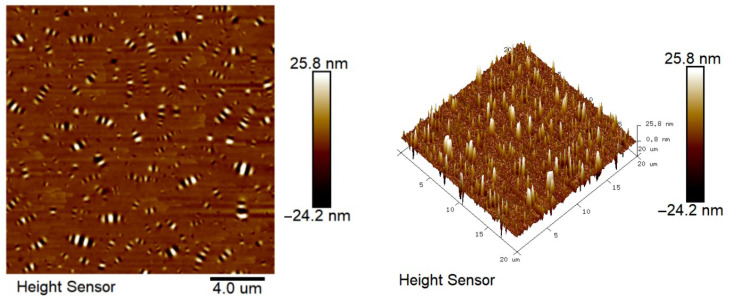
Micro-morphology of CF-modified asphalt.

**Figure 17:**
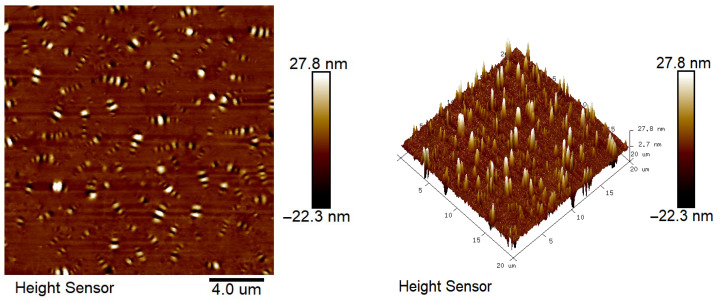
Microscopic morphology of PUP/CF-modified asphalt.

The results of atomic force microscopy of matrix asphalt, CF-modified asphalt, and PUP/CF composite-modified asphalt are shown in Figure 15, Figure 16 and Figure 17.

The sol–gel process is a key chemical method for synthesizing high-purity ceramic materials at lower temperatures than in conventional solid-phase reactions. The method facilitates precise control of composition, microstructure, and morphology, and sol–gel-derived ceramic fibers and matrices are critical in high-performance composites.

The hydrolysis sol–gel process involves a continuous reaction, with hydrolysis being the reaction of metal alcohol salts with water and polycondensation being the formation of M-O-M bonds from the hydrolyzed monomers via dehydration or dehydrogenation. The resulting colloidal suspension (sol) is transformed into a porous three-dimensional network (gel) suitable for oxide ceramics (SiO_2_, TiO_2_, Al_2_O_3_) and homogeneous doping and preparation of organic–inorganic hybrid materials. The non-hydrolyzed sol–gel process comprises direct condensation using the reaction between halides and oxygen donors or alcohol salts. An aluminosilicate (Al_2_O_3_-SiO_2_) fiber-reinforced polyurethane matrix can be prepared by using the sol–gel method. The selected polyurethane prepolymer is a partial reaction product of isocyanate and polyol, retaining the active -NCO group. With performance advantages, the three-dimensional network polyurethane structure is generated by a temperature-controlled curing reaction under the action of a chain-expanding crosslinking agent. The nanoscale uniformity of the aluminosilicate fibers optimizes the fiber/matrix interfacial bonding, the low defect rate of the sol–gel fibers significantly improves the fracture toughness of the composites, and the flexible network of the polyurethane matrix mitigates the interfacial failure caused by thermal stress.

From Figure 15, Figure 16 and Figure 17, it can be seen that the microscopic morphology of asphalt shows ‘bee-like structures’ with different depths and alternating black and white. These ‘bee-like structures’ are the aggregates formed by asphaltenes in asphalt through van der Waals forces and π-π stacking to form a rigid skeleton. The resin and saturates/aromatics are filled as dispersion medium to form a soft-phase region. The structure is shown as a convex light-colored area in the phase diagram, and the color of the surrounding depression is darker, reflecting the viscoelastic differences in different regions. CF modified asphalt has more small-sized ‘bee-like structures’ compared to matrix asphalt, and the dispersion is more uniform. This is because the addition of CF changes the rheological properties of asphalt, increases its viscosity and elasticity, decreases the mobility of asphalt molecules, and enhances the interaction between molecules. Asphaltenes that may have formed large-sized ‘bee-like structures’ can now only form smaller ‘bee-like structures’. CF forms a three-dimensional network structure in asphalt, which limits the free movement of asphaltene molecules and forces them to arrange in a smaller space to form a more uniform ‘bee-like structure’. Compared with CF-modified asphalt and matrix asphalt, PUP/CF composite-modified asphalt has more ‘bee-like structures’, which is because the unique molecular structure and properties of polyurethane prepolymer interact with asphalt molecules and change the asphalt, indicating that the gel-like polyurethane network is uniformly distributed within the asphalt matrix, contributing to a more stable structural asphalt phase.

### 2.8. Infrared Spectrum Test Analysis

**Figure 18:**
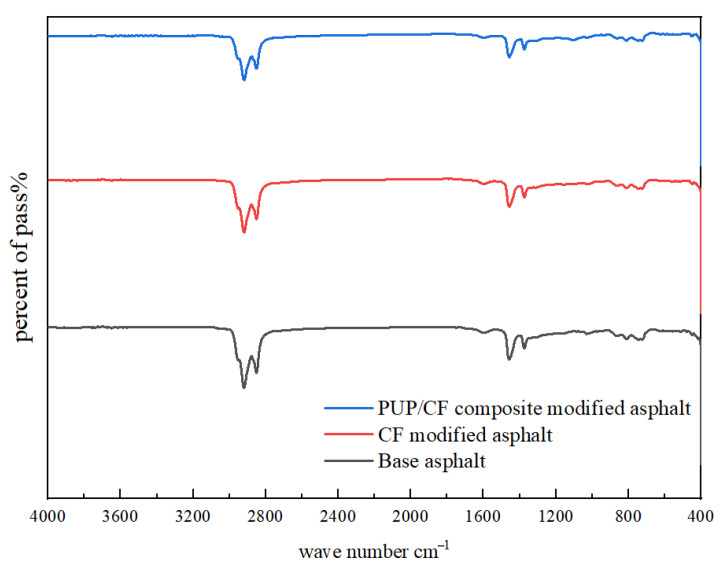
Infrared spectra of asphalt.

The test results of matrix asphalt, CF-modified asphalt, and PUP/CF composite-modified asphalt are shown in Figure 18.

The infrared spectrum of matrix asphalt in Figure 18 shows that there are two significant characteristic absorption peaks at 2919 cm^−1^ and 2851 cm^−1^, which are related to the stretching vibration of methyl (-CH) and methylene (-CH_2_-). A characteristic peak with low intensity appears at 1601 cm^−1^, which is usually related to the C=C stretching vibration of aromatic hydrocarbons. Medium-intensity stretching vibration peaks appear at 1455 cm^−1^ and 1372 cm^−1^, which are related to the bending vibration of methylene (-CH_2_-) and the symmetrical bending vibration of methyl (-CH_3_-). From 867 cm^−1^ to 721 cm^−1^, there are multiple absorption peaks related to the out-of-plane bending vibration of the C-H group of aromatic compounds. The infrared spectrum of CF-modified asphalt is basically the same as that of matrix asphalt, indicating that there is no chemical reaction between CF and asphalt. The peak intensity of PUP/CF composite-modified asphalt increases at 1595 cm^−1^. Because the benzene ring structure in polyurethane prepolymer enters the asphalt system through chemical bonding or physical blending, the peak intensity is enhanced. The PUP molecule has an isocyanate (-NCO) active group, and no absorption peak is found at 2270 cm^−1^, because it is related to the carboxyl group (-COOH) and hydroxyl group (-OH) in the asphalt.

## 3. Conclusions

(1) The three-factor and three-level optimization test was designed using the response surface method, and 17 groups of modified asphalt preparation schemes were created. The penetration, softening point, and ductility were used as response values. The influence of each factor on the response values and the interactions between each influencing factor were analyzed. The preparation scheme was optimized, and the optimal preparation scheme for composite-modified asphalt was determined. The polyurethane prepolymer content was 7.4%, the ceramic fiber content was 2.1%, and the shear time was 40 min.
(2) PUP/CF composite-modified asphalt exhibits the largest residual penetration ratio before and after aging, along with the smallest mass loss and softening point increment, indicating that PUP and CF enhance asphalt’s aging resistance, leading to superior aging performance. Its rutting factor is higher than that of CF-modified asphalt and matrix asphalt, showing better anti-rutting ability. Based on the time–temperature equivalence principle, the fitted complex shear modulus–frequency curve and viscoelastic master curve (using displacement factors) reveal that it has better high-temperature performance under low-temperature and high-frequency or high-temperature and low-frequency conditions.
(3) PUP/CF composite-modified asphalt has the best recovery strain capacity under low stress. At high stress levels, the recovery strain capacity of PUP/CF composite-modified asphalt is weakened, but the cumulative strain is significantly lower than that of matrix asphalt. Unlike previous studies focusing on single modifiers, this work combines gel-forming PUP with ceramic fibers (CFs) to form a hybrid microstructure, which not only results in significantly enhanced rheological and aging properties but also improves the flexibility and elasticity of composite-modified asphalt while enhancing its resistance to deformation.
(4) The stiffness modulus of PUP/CF composite-modified asphalt was significantly lower than that of matrix asphalt, which decreased by 35.1%, 49.1%, and 118.4% at −6 °C, −12 °C, and −18 °C, respectively. Compared with CF-modified asphalt, it is reduced by 11.8%, 13%, and 41.7%. The creep rate of PUP/CF composite-modified asphalt is large at three temperatures, indicating that PUP and CF have a good effect on the low-temperature rheological properties of asphalt.
(5) SEM observations show that carbon fiber (CF) is encapsulated by asphalt with minor voids which are filled by polyurethane prepolymer (PUP), strengthening CF–asphalt interfacial bonding via co-encapsulation for more stable performance. AFM micrographs reveal that PUP/CF composite-modified asphalt has more and larger ‘honeycomb-like structures’, indicating greater structural stability. Infrared analysis shows that PUP engages in hydrogen bonding and weak chemical reactions with asphalt, achieving modification through a synergistic combination of physical (predominant) and chemical means. This leverages PUP and ceramic fiber advantages: PUP physically encapsulates CF to reduce voids and enhance load transfer, and acts as a precursor to form a 3D elastic gel network that bridges CF–asphalt interfaces, boosting durability, mechanical resilience, and thermal stability under stress.
(6) In contrast to existing studies that investigate CF or PUP in isolation, this work focuses on the synergistic effects of gel-forming polyurethane prepolymers and ceramic fibers to enhance asphalt’s durability, stability, and resistance to deformation. The purpose is to develop a composite-modified asphalt suitable for extreme temperature conditions. Compared with base asphalt, the PUP/CF composite modification significantly improves tensile strength (by ~25%), fatigue life (by ~30%), and high-temperature rutting resistance (by ~20%) while also enhancing interfacial bonding and microstructural uniformity.

## 4. Materials and Methods

### 4.1. Matrix Asphalt

In this study, 70# road petroleum asphalt provided by Zhengfa Municipality of Zhengzhou City was selected as the base asphalt, and the basic properties of the base asphalt were tested according to the relevant regulations [44]. The test results are shown in Table 5.

### 4.2. Polyurethane Prepolymer

Polyurethane prepolymer is an intermediate product in the process of polyurethane synthesis which is formed by the partial reaction of isocyanate and polyol. It contains unreacted -NCO groups and can react with chain extenders to form polyurethane under certain temperature conditions. Under specific conditions, this polyurethane prepolymer reacts with asphalt components to form a three-dimensional crosslinked structure, exhibiting typical gel behavior. In this paper, the polyether polyurethane prepolymer produced by Jining Liduo Chemical Industry was selected, and its performance was determined according to the relevant specifications [45], as shown in Table 6.

### 4.3. Ceramic Fiber

The ceramic fiber (aluminum silicate) used in this study was purchased from Zhengzhou Xinyang Refractory Co., Ltd., with main components of Al_2_O_3_ (38–40%), ZrO_2_ (15–17%), and Fe_2_O_3_ (0.2%). The fibers were fabricated via melt-spinning. Its characteristics include high-temperature resistance, excellent chemical stability, and environmental protection. Its basic performance indicators are shown in Table 7.

### 4.4. Surface Modification of Ceramic Fiber

During the production, processing, or use of ceramic fibers, fiber particles may stick together or aggregate, forming larger clumps or agglomerates. This agglomeration phenomenon can adversely affect the performance of ceramic fibers, such as thermal conductivity, insulation, and mechanical strength. Therefore, surface modification treatment is performed on ceramic fibers to enhance their dispersibility and minimize agglomeration, thereby ensuring their stability and reliability. This paper used NaOH solution and a silane coupling agent (KH-550) to modify the surface of ceramic fibers cut into 6 mm lengths. The dispersion properties of the treated fibers were evaluated and analyzed. NaOH solution was selected because its strong alkalinity can remove impurities from the surface of ceramic fibers, roughen the surface, and introduce polar groups to improve wettability. Silane coupling agents were selected because their molecules can react with the hydroxyl groups on the surface of ceramic fibers and organic matrices at both ends, enhancing the compatibility between fibers and media through chemical bonding, thereby improving dispersibility and reducing agglomeration.

#### 4.4.1. Ceramic Fiber Modified by NaOH Solution

**Figure 19:**
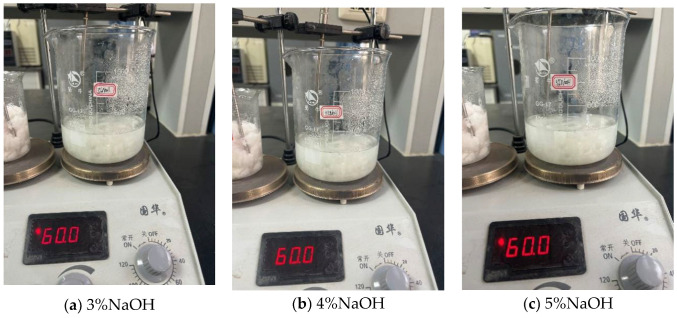
Modification with different concentrations of NaOH.

We prepared sodium hydroxide solutions with mass fractions of 3%, 4%, and 5%. We added ceramic fibers to sodium hydroxide solutions of different concentrations, placed them on a magnetic stirrer, heated them to 60 °C, and maintained a constant temperature for 0.5 h. As shown in Figure 19a–c.

#### 4.4.2. KH-550 Solution-Modified Ceramic Fibers

**Figure 20:**
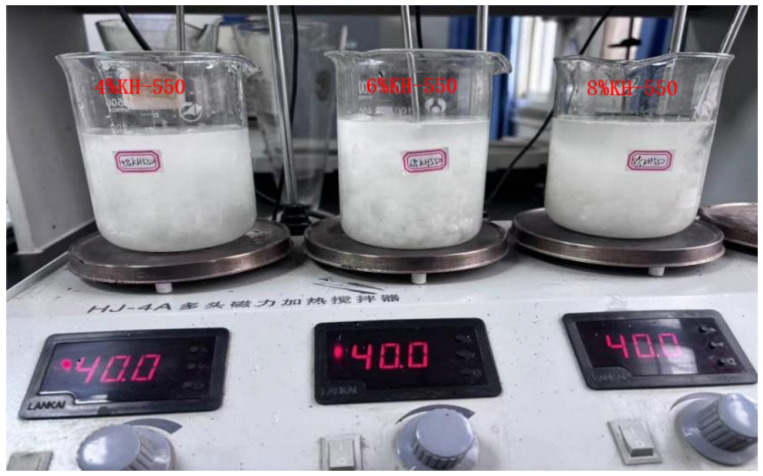
KH-55-modified ceramic fibers in different concentrations.

As shown in Figure 20, we mixed anhydrous ethanol and deionized water in a 2:8 ratio by weight to prepare the dissolving solution. We added 4%, 6%, and 8% coupling agents to the solution, stirred it evenly, and let it sit for 30 min to allow the silane coupling agent to hydrolyze fully. We put the ceramic fibers into different concentrations of KH-550 solution, heated them to 40 °C, and kept them at that temperature for 0.5 h.

### 4.5. MOCA

In this paper, 3,3-dichloro-4,4-diaminodiphenylmethane (MOCA) was selected. The chain-extending crosslinking agent MOCA can react with the isocyanate group in the polyurethane prepolymer to extend the molecular chain and form a three-dimensional network structure, thereby improving the mechanical, heat resistance, and chemical resistance of the polyurethane gel material. Its performance is shown in Table 8.

### 4.6. Test Scheme

#### 4.6.1. The Optimum Preparation of PUP/CF Composite-Modified Asphalt Based on Response Surface Methodology

According to the Box–Behnken design method in the response surface method, the experimental design of PUP/CF composite-modified asphalt was carried out by using the analysis software Design-Expert 13. The polyurethane prepolymer content (4~8%), ceramic fiber content (1~3%), and shear time (20~40 min) in the preparation process of modifying asphalt were selected as response variables. A three-factor and three-level optimization test was designed to study the effects of polyurethane prepolymer content, ceramic fiber content, and shear time on penetration (0.1 mm), softening point (°C), and 10 °C ductility (cm). A mathematical regression model between influencing factors and response values was established, and the optimal preparation scheme of composite-modified asphalt was determined.

#### 4.6.2. Rotating Film Oven Aging Test

The aging test of asphalt was carried out by using the rotating thin-film oven test (RTFOT). The asphalt sample was placed in a rotating thin-film oven and heated at a certain temperature. The rotating film can make the asphalt sample uniformly heated, ensuring that it is fully in contact with the air, and simulate the aging process in the actual environment. The thermal stability and volatility of asphalt can be evaluated by measuring the mass change, viscosity change, and softening point change of asphalt samples before and after heating.

#### 4.6.3. Dynamic Shear Rheological Test

Dynamic shear test (DSR) is an important test method for evaluating the rheological properties of asphalt and its modified materials under high-temperature and low-frequency conditions. This test is mainly used to determine the viscoelastic behavior of asphalt. Temperature scanning is an important test method for evaluating the rheological properties of asphalt with temperature changes. The strain level was 10%, the temperature range was 46~82 °C, the temperature interval was 6 °C, and the angular frequency was 10 rad/s. Frequency scanning is a test method for measuring the mechanical properties of viscoelastic materials such as asphalt at different frequencies. The test temperature was set at 40~88 °C, the temperature interval was 12 °C, the frequency range was 0.1~100 rad/s, and the strain level was controlled at 1%.

#### 4.6.4. MSCR Test

The MSCR test is based on the creep theory. During the test, a constant axial stress was applied to the asphalt and maintained for a certain time to cause creep deformation of the specimen. Then, the stress was unloaded, and the asphalt was allowed to recover for a certain period of time. The deformation of the specimen in each loading-recovery cycle was observed and recorded, including recoverable strain and unrecoverable strain. Repeated loading–recovery cycles were performed at two common stress levels of 0.1 kPa and 3.2 kPa. The loading time was 1 s, the unloading time was 9 s, and the number of cycles was 10 times.

#### 4.6.5. Bending Creep Stiffness Test

BBR evaluates the low-temperature rheological properties of asphalt by measuring the bending deformation of the beam sample at a certain temperature. The basic principle is that the asphalt sample is prepared into a long strip, and then a constant load is applied in the test. The low-temperature performance of the asphalt is judged by measuring the deformation of the beam in a certain period of time. In the test, PUP/CF composite-modified asphalt was tested at −6 °C, −12 °C, and −18 °C, and the stiffness modulus S and creep rate m were measured.

#### 4.6.6. Scanning Electron Microscopy Test

This study used a scanning electron microscope model JSM-7500F manufactured by Shanghai Bahe Instrument Technology Co., Ltd. (Shanghai, China). The test employed electron optics principles to emit a low-energy electron beam onto the asphalt surface. The electron beam interacts with the asphalt atom, and the generated signal is processed to generate an observation image. Different from ordinary microscopy, scanning electron microscopy can enlarge the material to nanometer scale and obtain detailed observations of the microstructure and distribution of the material. In the study of composite-modified asphalt, by adjusting different magnifications, the bonding state and distribution of the admixture and asphalt can be clearly observed, and the differences in the microstructure of the two can be distinguished so as to further analyze the modification mechanism of the modifier on asphalt.

#### 4.6.7. Atomic Force Microscope Test

Using the JSM-7610FPlus atomic force microscope provided by Jieouluo Trading Co., Ltd., it is possible to observe the nanoscale structure of the asphalt surface with a resolution of up to 0.1 nanometers. The microstructure of asphalt can be understood more deeply, and the modification mechanism can be analyzed. The scanning frequency of this test was 1 Hz, the scanning pixel size was 512 × 512, the image scanning area was 20 μm × 20 μm, the resolution was 10 nm, and the experimental temperature was 25 °C.

#### 4.6.8. Infrared Spectroscopy Test

A TENSOR 37 Fourier transform infrared spectrometer from Bruker Spectrometer Instruments was used. The scanning range as 4000~500 cm^−1^. By comparing the infrared spectra of asphalt before and after modification, the change in absorption peak can be observed. If a new absorption peak appears in the modified spectrum, it indicates that the modifier reacts with the asphalt to form a new functional group. If the position or intensity of some original absorption peaks change, it may mean that the chemical structure of the asphalt has been altered due to modification.

## Figures and Tables

**Table 1:** Design-Expert-optimized experimental design.

Serial Number	Polyurethane Prepolymer Content%	Ceramic Fiber Content%	Shear Time Min	Penetration	Softening Point	Ductility
1	4	1	30	42.7	52.1	18.5
2	8	1	30	40.5	53.3	20.2
3	4	3	30	38.1	55.6	17.6
4	8	3	30	36.5	56.5	19.6
5	4	2	20	40.6	54.6	18.9
6	8	2	20	38.7	55.7	20.8
7	4	2	40	40.0	55.1	19.5
8	8	2	40	37.8	56.0	21.0
9	6	1	20	42.2	52.7	19.5
10	6	3	20	38.8	56.2	18.7
11	6	1	40	41.2	53.0	20.1
12	6	3	40	37.3	56.4	19.8
13	6	2	30	38.2	55.4	20.9
14	6	2	30	38.5	55.5	20.8
15	6	2	30	39.1	55.7	20.9
16	6	2	30	38.7	55.6	21.2
17	6	2	30	38.5	55.5	20.9

**Table 2:** Optimization value of preparation parameters.

Preparation Parameters	PUP (%)	CF (%)	Shearing Time (min)
optimal value	7.4	2.1	40

**Table 3:** Properties of the composite modified asphalt with the optimal dosage.

Indicators	Needle Penetration (0.1 mm)	Softening Point (°C)	Ductility (cm)
Predicted value	38.6	55.54	20.94
Measured value	38.2	56.2	20.4

**Table 4:** Asphalt RTFOT indicators before and after aging.

Performance Index	Matrix Asphalt		CF-Modified Asphalt		PUP/CF Composite-Modified Asphalt	
	Before Aging	After Aging	Before Aging	After Aging	Before Aging	After Aging
Mass (g)	35.140	35.018	35.312	35.258	35.374	35.344
Penetration at 25 °C (0.1 mm)	66.1	53.7	55.9	40.5	40.6	34.7
Softening Point (°C)	50.8	53.7	53.3	56.4	57.5	60.2
Mass Loss (%)	0.348		0.152		0.086	
Penetration Ratio (%)	69.6		72.5		85.4	
Softening Point Increase (°C)	2.9		2.1		1.7	

**Table 5:** Basic performance index of asphalt.

Performance Index		Test Results	Unit	Technical Requirements
Penetration (25 °C)		66.1	0.1 mm	60~80
Softening point		50.8	°C	≥46
Ductility (5 cm/min, 10 °C)		30.2	cm	≥20
Flash point		270	°C	≥260
Density (15 °C)		1.136	g/cm^3^	measured value
After aging	Quality change	−0.122	%	≤±0.8
	Softening point increment	2.9	°C	≤8 °C
	Penetration ratio	69.6	%	≥61

**Table 6:** Basic properties of polyurethane prepolymers.

Performance Index	Unit	Test Results
NCO content	(%)	5.1 ± 0.2
State	/	Colorless transparent liquid
Viscosity	(85 °C/MPa.s)	350
Hardness	Shore A	90 ± 2
Tensile strength	(MPa)	30
Elongation at break	(%)	420
Rebound	(%)	32

**Table 7:** Basic performance indicators of ceramic fiber.

Performance Index		Ceramic Fiber (Aluminum Silicate Fiber)
Classification Temperature (°C)		1400
Diameter (m)		3–5
Slag ball content (%)		≤15
Chemical composition (%)	Al_2_O_3_	38–40
	Fe_2_O_3_	0.2
	ZrO_2_	15–17

**Table 8:** Basic performance of MOCA.

Performance Index	Appearance	Molecular Weight	Melting Point (°C)	Structural Formula
Test results	Yellow particles	267.16	105	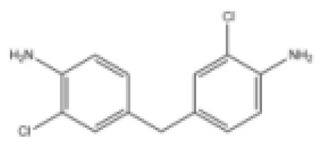

## Data Availability

Some or all of the data, models, or code that support the findings of this study are available from the corresponding author upon reasonable request.
